# Seasonal and daily protandry in a cyprinid fish

**DOI:** 10.1038/s41598-017-04827-x

**Published:** 2017-07-05

**Authors:** Marek Šmejkal, Daniel Ricard, Lukáš Vejřík, Tomáš Mrkvička, Lucie Vebrová, Roman Baran, Petr Blabolil, Zuzana Sajdlová, Ivana Vejříková, Marie Prchalová, Jan Kubečka

**Affiliations:** 1 Institute of Hydrobiology, Biology Centre of the Czech Academy of Sciences, České Budějovice, Czech Republic; 2 Faculty of Science, University of South Bohemia, České Budějovice, Czech Republic; 3 Faculty of Economics, University of South Bohemia, České Budějovice, Czech Republic

## Abstract

In polygynandrous mating systems, in which females limit reproductive success, males can increase their success by investing in courtship. Earlier arrival at the spawning ground compared to when females arrive may increase their opportunities in competitive mating systems. In this study, we used passive telemetry to test whether a male minnow known as the asp, *Leuciscus aspius*, times its arrival at spawning grounds relative to the arrival of females. Males arrived in a model stream approximately five days earlier than females on average and left four to five days later than females over two years. Both sexes performed a daily migration between a staging ground (standing water, low energy costs) and the fluvial spawning ground (high energy costs). Fish abundance peaked twice a day, with a major peak at sunset and a minor peak at sunrise and with the evening peak abundance for males occurring 1 hour 40 minutes earlier than that of females. The number of females on the spawning ground never exceeded the number of males. While the degree of protandry is hypothesized to be influenced by the operational sex ratio (ranging from 0.5 to 1 in our study), our data did not support this theory.

## Introduction

Successful reproduction at least once in each individual’s lifetime is crucial for every living organism. Sex-dependent chances of reproduction vary among mating systems and types of parental care^1, 2^. The most intense competition for mates occurs in the sex that make up the majority of the breeding population and has the higher reproductive potential^3, 4^. In most animal species, females are the sex with the limiting reproductive rate, which favours male intra-sexual competition and active courtship^5, 6^. Strong male competition is especially predominant in mating systems in which males do not participate in parental care or feed females^7–9^.

In polygynandrous mating systems, males typically exhibit high levels of mate-finding activity to increase their reproductive success^5^. Reproductive gatherings in certain areas help males in their search for females; however, male intra-sexual competition is intense in such systems^10, 11^. Examples of reproductive gatherings in which male success differs considerably among individuals include lek systems and many fish spawning grounds^12–14^. In these mating systems, male behaviours that increase the probability of successful mating are under strong sexual selection^15^.

Although there are a wide variety of types of reproductive gatherings, we still see common patterns among different mating systems. In species in which mating occurs at the gathering site and that have conventional sex roles, males exhibit protandry, generally arriving at the breeding ground before females during the breeding season^16–18^. This early arrival is used to establish intra-sexual hierarchies and territories in many types of reproductive gatherings and lek mating systems, and thus, early arriving males may achieve a higher rank and consequently have access to more females (rank advantage hypothesis)^16, 19, 20^. Alternatively, protandry may simply result in maximizing the number of mating opportunities by merely allowing males to encounter a higher number of females during the mating season (mate opportunity hypothesis)^21, 22^. While protandry has positive effects on male fitness, early arrival can reduce survival because harsh environmental conditions are common at the beginning of the mating season^21, 23, 24^. Finally, a male-biased operational sex ratio should generally promote protandry, as the competition for mates is very high in male-dominated systems^22^.

The length of the ready-to-mate period is usually sex-specific. While males are prepared to breed over a relatively long period in a given season, female receptivity determines when mating actually occurs^25, 26^. Therefore, a possibly beneficial strategy for males is to always be ready and wait for the chance to mate^21, 22, 27^. However, daily energy expenditure by males is high during the reproductive period. In male Atlantic salmon, *Salmo salar*, the energetic costs of mating counterbalance the six-times-higher investment in gonadal development by females^10, 28^. Hence, if the mere presence at the breeding site is costly in comparison to the cost of remaining in the surrounding environment (e.g., due to male competition or a hostile environment), the allocation of energy by males should be aimed at the specific seasonal and daily periods when the chances of encountering a receptive female are the highest^29^. Furthermore, daily male arrival should be dependent on the current operational sex ratio, and daily protandry should occur especially when the sex ratio is strongly male-biased^17, 22^.

Here, we studied the sex-specific seasonal and daily aspects of the timing of reproductive migrations in an iteroparous fish, *Leuciscus aspius* (asp), Cyprinidae, inhabiting European lentic and lotic environments. The asp spawn following a polygynandrous mating system typical for the majority of cyprinid species, and its spawning grounds are restricted to fast flowing rivers^30, 31^. Males actively search for receptive females in the spawning grounds. The act of spawning consists of one or multiple males vigorously chasing a female and terminates in the simultaneous release of eggs and milt near the surface of the water. The negatively buoyant, adhesive eggs drift in the water current and eventually stick to the stony ground. Asps do not form permanent pairs during spawning, which results in an open contest for receptive females^32^. There is no parental care, i.e., reproductive costs only involve gonadal development and the energetic costs associated with migration to and movement at the spawning grounds. In the system we studied, the entire reproductively active population migrates daily from the staging ground in a reservoir (standing water, low energy costs) to the spawning ground in a short tributary (fast-flowing water, high energy costs). Such a system represents an ideal model for the study of both seasonal and daily aspects of protandry because all individuals migrate to a space-limited spawning ground ideal for passive telemetric monitoring. In this study, monitoring tiles were used to ensure that the spawning ground is well-defined and the asp do not spawn below the monitoring site. In order to test the male and female difference in timing of arrivals and departures on the spawning ground, 433 males and 316 females were captured and individually tagged with passive integrated transponders (PIT tags) in 2014 and 2015. In subsequent spawning seasons, passive telemetry systems were installed in spatially delimited spawning ground for the month-long spawning season and altogether, 351 tagged individuals from previous years were detected. Timing of individual arrivals and departures on the spawning ground were recorded by the passive telemetry systems.

The goals of this study are specifically to test I) whether males exhibit protandry in their seasonal and daily migration, II) whether males also leave later (both on a daily and seasonal basis) than females, III) whether males time their daily length of stay to the number of females present and IV) whether the degree of daily protandry correlates with the daily operational sex ratio.

## Results

### Monitoring coverage of the spawning ground

**Figure 1:**
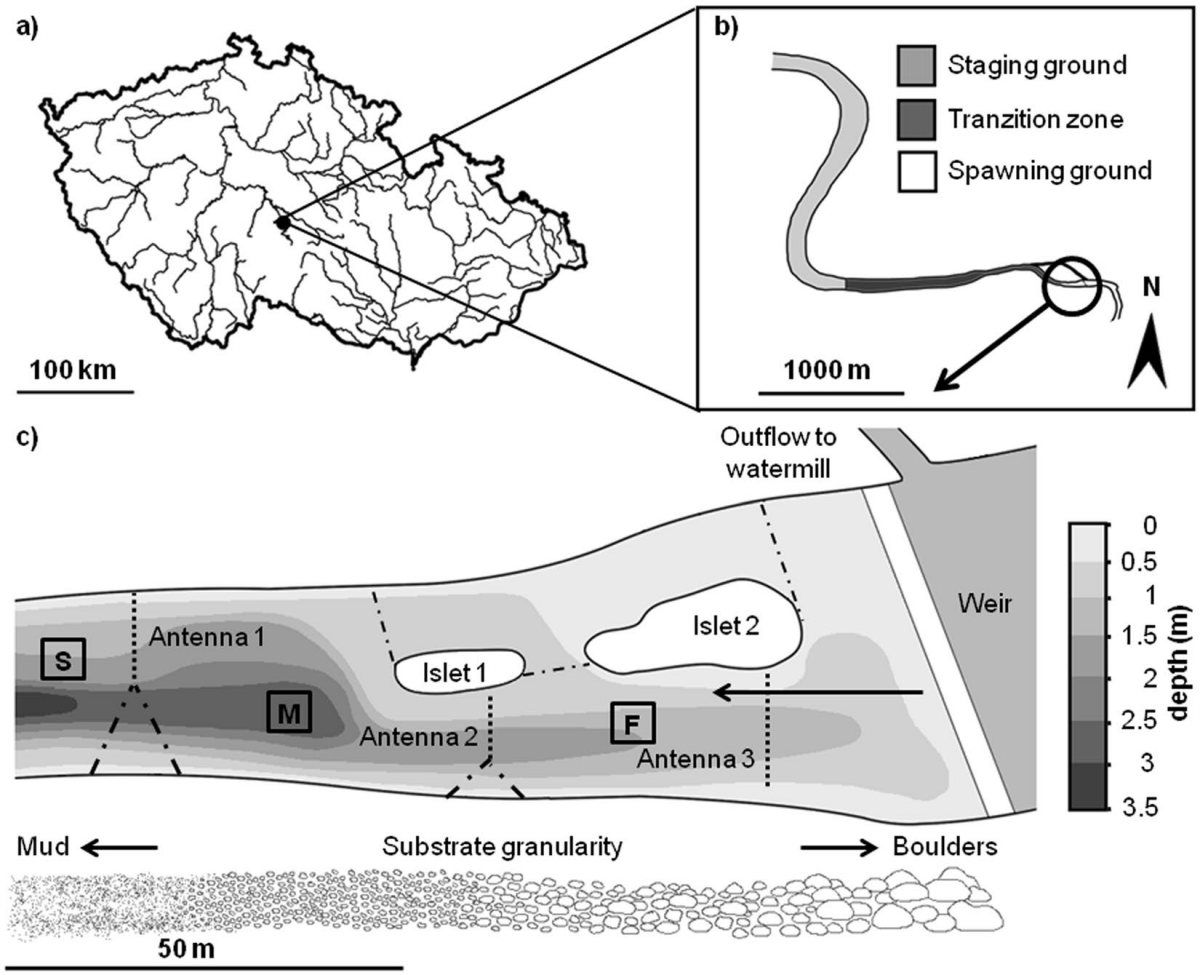
Schematic representation of the position of Želivka Reservoir in the Czech Republic (**a**), approximate position of staging ground and transition zone trough which fish migrate (**b**), and detail of monitored spawning ground with the experimental setup (**c**). Placement of antenna systems is indicated by dotted lines. Antennas 1 and 2 were deployed in 2015–2016 and the additional antenna 3 in 2016. Nets guiding asps into antennas are indicated by dot-dashed lines. Squares S, M and F represent sites for asp egg monitoring in 2016 where 4 tiles were placed at each site (S - slow, M - medium and F – fast current). Arrow shows the direction of flow. The figure was generated by the software ArcMap, version 10.2.2^53^.

The monitoring sites were significantly different, and the uppermost egg monitoring site was the most used spawning ground (likelihood ratio test: χ^2^ (df = 2) = −359.8, p < 0.001; mean number of eggs ± SD - fast 70 ± 68, medium 40 ± 46, slow 1 ± 1). Furthermore, the single likelihood tests demonstrated that the slow site was significantly different than the fast site (Wald test: χ^2^ (df = 1) = 73.4, p < 0.001) and the medium site (Wald test: χ^2^ (df = 1) = 32.4, p < 0.001). Based on these results, we conclude that studied reproducing asps had a spatially delimited spawning ground and that the spawners had to pass at least one antenna to enter the spawning ground (Fig. 1).

### Seasonal protandry, day of leaving and movement at the spawning ground

**Figure 2:**
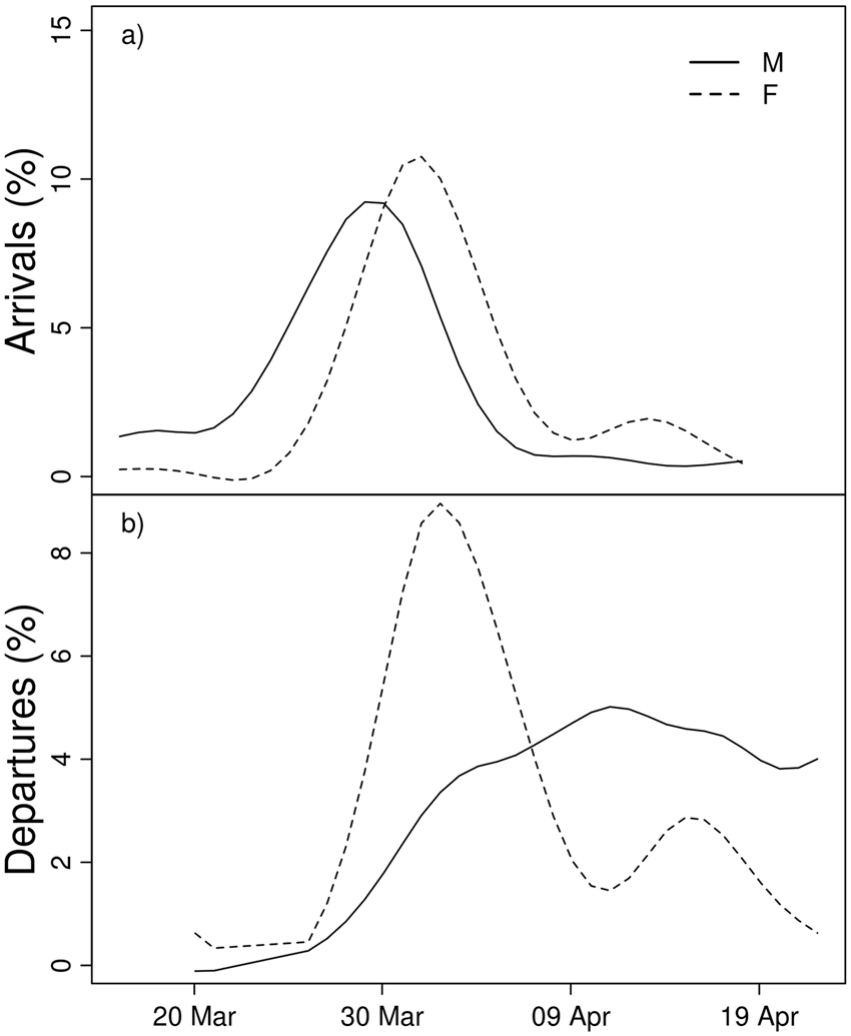
Arrival (**a**) and departure dates (**b**) in 2016 plotted for females (F) and males (M) separately. Lines in panels (**a**) and (**b**) represent the percentage of arriving and departing individuals in a given day. Raw data are provided in Supplementary Fig. S1.

Ninety-nine tagged males and 51 tagged females were detected by the antennas in 2015, and 171 males and 101 females were detected in 2016. When present on the spawning ground, males were detected approximately twice as frequently per hour than were females, indicating their higher level of mate-finding activity. Generally, males invested approximately three times as much time to spawning as females in both years (in terms of both hours and days). When measured from the first day of monitoring in a given year, males arrived for the first time at the spawning ground on average five days earlier and left four to five days later than females (Table 1, Fig. 2). The male-female comparisons were almost identical in both seasons despite the fact that we increased the precision of monitoring by deploying a third antenna system in the spawning ground.

**Table 1:** Summary of movement at the spawning ground, seasonal protandry and times of leaving in the 2015 and 2016 seasons. Mean values, medians, first (Q_1_) and third quartiles (Q_3_), values from the one-sided Wilcoxon rank-sum test, sample sizes (N) and p-values are presented in the table. Significant p-values are given in bold.

Variable	Season	Mean		Median		Q_1_		Q_3_		Wilcoxon	N	P
		M	F	M	F	M	F	M	F			
Detections per hour	2015	27	15	18	15	14	12	22	17	1640.0	150	<**0.001**
	2016	55	25	28	25	22	19	46	30	6342.0	272	<**0.001**
Hours	2015	44	17	33	17	16	11	70	23	1331.5	150	<**0.001**
	2016	67	17	53	14	31	9	90	22	1928.0	272	<**0.001**
Days	2015	10	3	9	3	6	2	14	4	798.5	150	<**0.001**
	2016	11	3	10	2	7	2	14	3	1563.0	272	<**0.001**
Arrival day No.	2015	7	13	6	15	2	7	13	17	3794.0	150	<**0.001**
	2016	14	19	14	17	11	16	16	20	13154.0	272	<**0.001**
Departure day No.	2015	20	16	19	17	17	16	22	18	1460.5	150	<**0.001**
	2016	27	22	27	20	22	17	33	24	4788.0	272	<**0.001**

### Daily protandry, time of leaving, male length of stay and operational sex ratios

**Figure 3:**
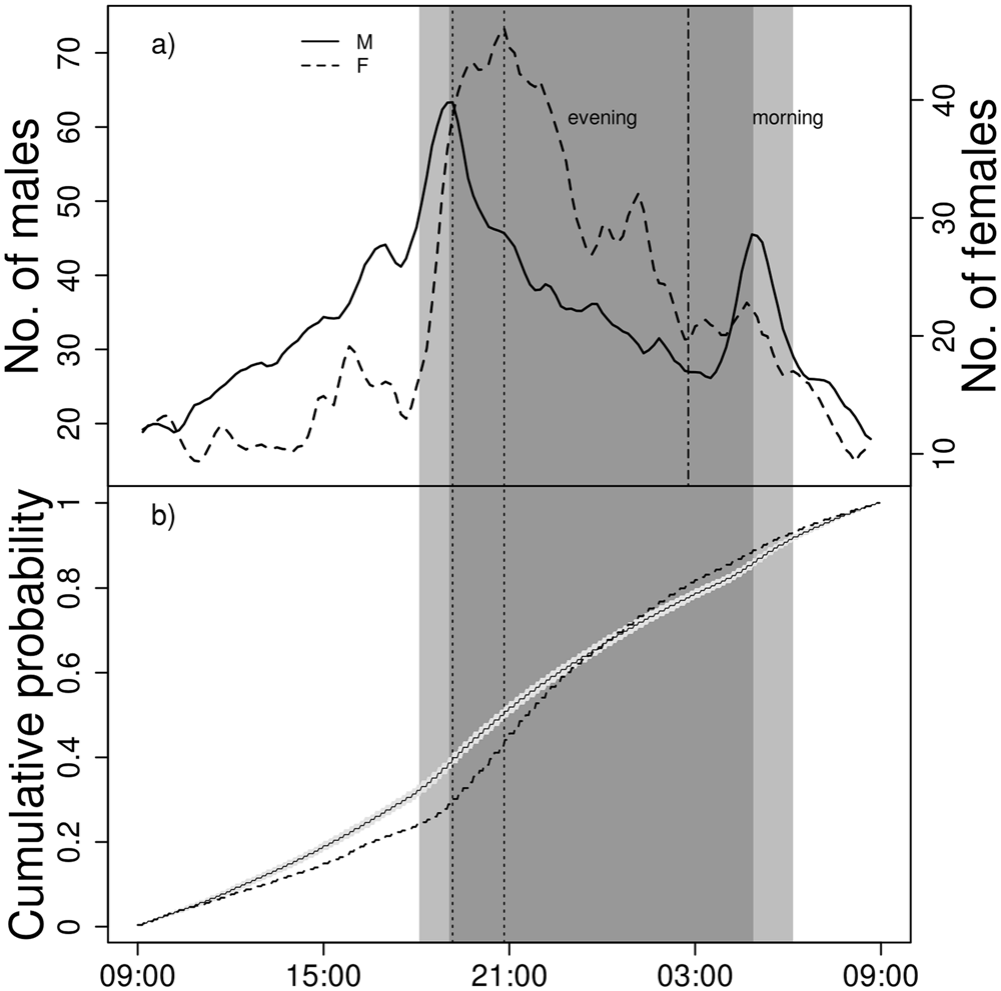
The male and female presence on the spawning ground in 24-hour cycles starting at the lowest observed attendance (**a**), and the rank envelope test comparing general fish presence with female presence at the spawning ground (**b**). In the upper panel, the weighted male and female curves were standardized to the same height for better comparison. Moving average with 30-minute interval was used for the plot. Male and female peaks of presence are indicated by dotted lines. Division between morning and evening period defined as points with the lowest abundance is indicated by dot-dashed line and by starting time of the plot. In the lower panel, any departure of the data from the global envelope proves the rejection of the null hypothesis that the female curve is the same as male curve. The light grey area demonstrates the shift between sunset and sunrise during the monitoring period.

The distributions of the daily presence of males and females were significantly different (Rank envelope test, N = 351, p = 0.005), indicating that more males than females arrive before sunset and more females than males arrive at night in relative numbers (Fig. 3).

**Figure 4:**
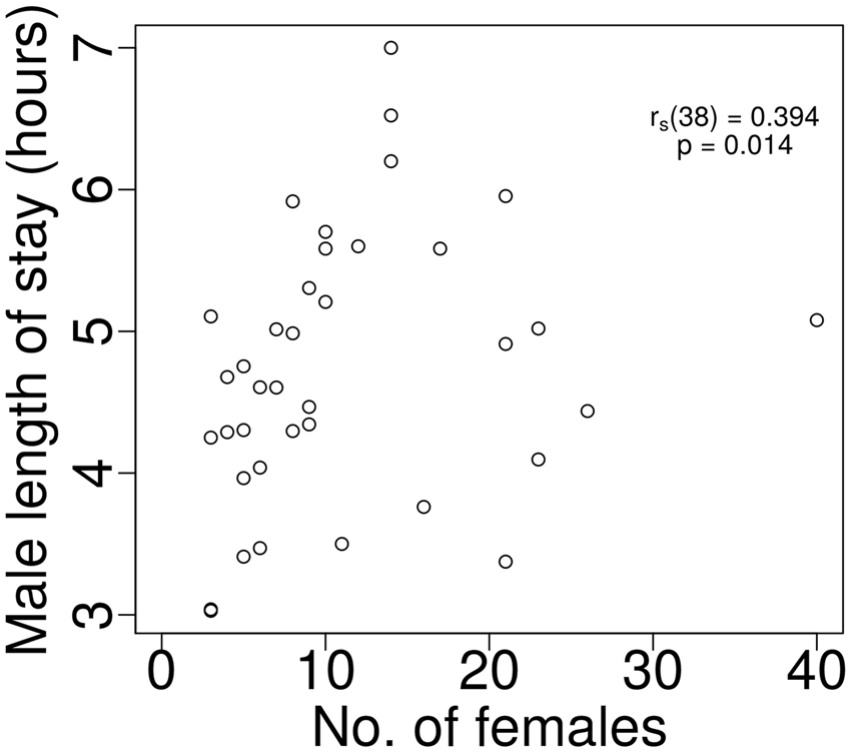
The relationship between male length of stay and the number of females on the spawning ground in the evening spawning period.

A Spearman’s rank-order correlation revealed a positive relationship between male length of stay and number of females on the spawning ground in the evening period (r_s_ (38) = 0.394, p = 0.014; Fig. 4), while the relationship was not significant in the morning period (r_s_ (24) = 0.234, p = 0.271).

The operational sex ratio was male-biased during both the evening and morning periods (evening median = 0.79 with interquartile range IQR = 0.70–0.88, morning median = 0.88, IQR = 0.73–1). Male daily protandry was not correlated with the operational sex ratio during either the evening or morning period (evening period: GLM ratio F = 0.21; df = 1; p = 0.649; morning period: GLM ratio F = 0.01; df = 1; p = 0.952).

## Discussion

Currently, sex-dependent reproductive timing in fish has been studied mostly in species with a certain level of parental care^10, 26, 33–35^, whereas investments of species spawning in aggregations with no parental care have not received much scientific attention. However, these mating systems are very suitable for testing protandry – there is no permanent pair formation, and therefore, males should maximize the number of spawning events and the number of acquired females as the reproductive success of males depends solely on the number and quality of eggs he fertilizes^36^. From this point of view, mating opportunities should increase more steeply with increasing protandry and reproductive activity than in species in which pairs are formed early in the season and in which surplus paternity can be achieved only by mating with subsequently acquired females during the season or via extra pair paternity^37, 38^. In our study, high male investments into reproduction are demonstrated by their length of stay and by their greater movement at the spawning ground.

Over the course of the spawning season, asp males preceded females by five days on average in their seasonal arrival time and left four to five days later. Because fish sex can only be ascertained with certainty during the spawning season^39^, individual fish were tracked in the spawning season following tagging to avoid any influences of electrofishing and manipulation on their spawning behaviour. Although this enabled us to precisely analyse the sex-specific differences in both seasonal and daily arrival times, the analyses are biased towards experienced individuals as their first spawning season could not be monitored because of the nature of our methodology. Young individuals may not time their arrival to the spawning ground as precisely as experienced fish, as has been demonstrated in pike, *Esox lucius* ^40^. The tagging of juveniles and subsequent sex determination based on behaviour at the spawning ground may shed light on any differences between inexperienced and experienced spawners.

The longer period of activity at the spawning ground by males should favour protandry, as has been assumed in a model for salmon^41^. This requirement of protandry is fulfilled in the asp mating system, in which males stay at the spawning ground for 10 days on average, which is three times longer than females stay. Male departure is most likely determined by exhaustion and a lack of milt. We do not think that protandry in asps is driven by the differential susceptibility hypothesis, which postulates that the larger sex arrives first due to its greater resistance to harsh environmental conditions^42^. Asp males are generally smaller than females (see the lengths of tagged males and females in the methods section), and hence, the larger female body size would predict that females, not males, should arrive earlier in the spawning season. Instead, we believe that sexual selection drives the male arrival strategy and their length of stay at the spawning ground.

Research on seasonal protandry has provided evidence that this phenomenon may positively affect male fitness in several ways. Protandry may result in a higher number of sired offspring because early-arriving females are commonly in prime body condition and may produce more offspring than late-arriving females^11, 43^. In mating systems where pairs are formed early in the season, the early-arriving male may form more than one pair in the mating season^21^ or may achieve more offspring via extra-pair paternity^37^. Additionally, early-acquired offspring may have higher chances of survival, possibly giving the offspring higher chances of being recruited into the breeding population^44^. Furthermore, greater seasonal protandry should be favoured when female arrival is protracted^41^. This was indeed the case in our study, which showed that the spawning season lasted for approximately one month in both years, while the average female spent only three days in the spawning ground. However, we do not have individual mating success data, and hence, further research is needed to reveal the consequences of early seasonal arrival for individual males.

Protandry may have positive effects on fitness not only seasonally but also daily. In Dawson’s burrowing bees, males are active early in the day and search for later-emerging females^45^. In the asp mating system studied here, the proximity of standing water and the requirement of a fluvial spawning ground presents an energetic trade-off, which likely results in daily migration between the spawning ground, with its associated high energetic costs, and the staging ground located in the standing water, with its lower associated energetic costs. Males arriving earlier to the spawning ground may encounter more opportunities to mate with newly ovulating females. Although we have no data on the duration of ovulation in females, many females spent very little time in the spawning ground, suggesting that this period was most likely very closely related to the actual female spawning time. Hence, it would indeed be advantageous for males to arrive before females on a daily basis. Our data demonstrate that males arrived earlier in the evening peak of asp spawning activity, and males stayed longer in the morning than females. Although the daily data may suggest a certain mismatch between male and female presence, we have to emphasize that the actual estimated number of females never exceeded the number of males at the spawning ground.

The daily pattern of fish presence, with evening and morning peaks, may be driven by internal mechanisms similar to those found to synchronize spawning in goldfish *Carassius auratus* reviewed in Sorensen & Wisenden^36^. In the goldfish mating system, fish are reported to spawn from morning to noon, whereas asp spawning activity was observed throughout day and night, with an uneven number of spawners and with the highest effort around sunset and sunrise. Asp females spent an average of six hours at the spawning ground and paid it three visits, possibly temporarily running out of eggs ready for ovulation during each visit. The relative synchrony of these female visits seemed to create the distinct peaks of female spawning activity. Hence, male timing should take into account the high number of mating chances at these hours but should also consider the other periods of the day where there are more limited chances to mate.

While males adjusted their length of stay according to the number of females in the spawning ground, the actual increase in time was not very large and was only significant in the evening period. We assume that males were limited by milt storage in their sperm ducts during the periods with many females due to excessive mating and eventually returned to the staging ground to restore the necessary amount of milt^46^.

Because fish capture was strongly dependent on their reproductive activity (fish captured in the staging ground were present or future active spawners), our day-to-day catches reflect the operational sex ratio better than the adult sex ratio in the asp population. Due to the very short-term presence of females compared to that of males, the adult sex ratio could be more reliably assessed only based on recaptures in the following spawning season. Hence, the adult sex ratio could be estimated only for 2014 and 2015, when it was 0.47 and 0.42, respectively^32^. However, females are likely to skip a spawning season^9, 47^, which may consequently cause the overestimation of the number of females in the system due to their relatively lower recapture rate compared with that of males. For these reasons, we instead used the operational sex ratio in our analyses.

## Conclusions

In animal species in which the male has a higher potential reproductive rate and does not provide any parental care, it is the male who has to invest more energy in courting behaviours. Our study presents field evidence of the seasonal and daily aspects of protandry in asps. To our knowledge, this study provides the first evidence of daily protandry in a vertebrate, the extent of which seems not to be driven by changes in the operational sex ratio at the breeding ground.

## Materials and Methods

### Study site

The study was conducted in the largest water-supply reservoir in Central Europe, Želivka Reservoir, which is located at 49°57′84″ N, 15°25′16″ E in the Czech Republic. The reservoir has one large inlet, the Želivka River. A weir located on the river just above the reservoir blocks further upstream migration and restricts asp spawning to a 100-m long and, on average, 22-m wide stream site (Fig. 1). The bottom of the transition zone between the standing water and the upstream spawning site consists of clay deposits that are unsuitable for asp spawning and egg development.

### Fish capture, tagging and migration recording

Individual spawning asps were captured at the staging ground using an electrofishing boat (electrofisher EL 65 II GL DC, Hans Grassel, Schönau am Königsee, Germany, 13 kW, 300/600 V) from 26 March to 3 April 2014 and from 4 to 11 April 2015. The fish were anaesthetized with benzocaine, and their total length (TL), sex and weight were recorded. The males were distinguished based on milt release during the tagging period, breeding tubercles and a slender body, whereas females had no tubercles, a robust body and predominantly released eggs at the end of the tagging period. After removing 3–4 scales, a 4–5 mm vertical incision was made 3 cm posterior to the pelvic fin, and a PIT tag (Oregon RFID, half-duplex, length 32 mm, diameter 3.65 mm, weight 0.8 g, ISO 11784/11785 compatible) was inserted into the body cavity. No sutures were used to close the incision, according to a previously described cyprinid tagging methodology^48, 49^. The tagged individuals were released immediately after recovery from anaesthesia. Altogether, 221 males (597 mm TL ± 53 mm SD) and 135 females (635 ± 75 mm) were tagged in 2014, and 212 males (565 ± 42) and 181 females (595 ± 62) were tagged in 2015. The tagged individuals represented a substantial part of the mature asp population in the studied reservoir; the population size of spawning individuals, based on mark-recapture estimates, was 979 males and 1107 females in 2014^32^.

The movements of the tagged individuals were monitored via passive telemetry using Oregon RFID antenna systems (LF HDX RFID readers). The PIT tag is energized each time a fish passes the antenna and then emits an individual code that is recorded and stored together with the date and time. Two (three in 2016) RFID antennas were installed in the main tributary of the Želivka Reservoir 50 m apart (Fig. 1), and their time settings were synchronized. The first and second antenna systems were installed in the downstream and middle sections of the spawning ground, respectively, and in 2016, a third antenna was installed in close proximity to the weir to increase the coverage and probability of fish detection at the spawning ground. The river topography allowed us to only cover half of the river, so we guided the asp to swim through the 10-m wide antennas using nets with a 40-mm mesh size (Fig. 1). Fast currents prevented the installation of guiding nets and their attachment to the third antenna, so one guiding net was attached to the island only, and hence, fish could swim close to the left bank of the river without being detected. The system recording frequency was set to 10 energize/receive cycles s^−1^, and the systems were tested daily to ensure that they were scanning their entire detection range. Monitoring began prior to the beginning of asp spawning (25 March 2015 and 16 March 2016) and continued until the majority of the asp disappeared from the spawning ground (21 April 2015 and 22 April 2016).

### Monitoring of asp eggs

We monitored egg density on standardized concrete tiles (Fig. 1) to delimit the size of the spawning ground in 2016. A hole was drilled in the centre of each tile, which was then attached to a rope with a float to facilitate monitoring. At three sites in the spawning ground, a total of 12 concrete tiles (40 × 40 cm) were deployed. The surrounding spawning substrate consisted of large stones and pebbles. The depth and length profile was measured at each site, and water flow was estimated as fast (34 ± 9 cm s^−1^), medium (27 ± 7 cm s^−1^) or slow (16 ± 4 cm s^−1^) for each egg monitoring site based on the daily total water flow data recorded by the river authority (Povodí Vltavy). The tiles were checked daily for new eggs at 8 am and 7 pm, and eggs were removed after being counted.

The field sampling and experimental protocols used in this study were performed in accordance with the guidelines of and with permission from the Experimental Animal Welfare Commission under the Ministry of Agriculture of the Czech Republic (Ref. No. CZ 01679). All methods were approved by the Experimental Animal Welfare Commission under the Ministry of Agriculture of the Czech Republic.

### Data analysis

#### Monitoring coverage of the spawning ground

We first tested whether asps had to pass at least one antenna to reach the main spawning ground. The counts of new eggs from the three monitoring sites (Fig. 1) were used as a sign of the extent of the spawning ground, and the total number of eggs on each tile was used for the statistical analysis. Among-site comparisons were then performed using a generalized linear model assuming that egg numbers followed a Poisson distribution. The logit link was used in the Poisson model.

#### Seasonal protandry, day of leaving and movement at the spawning ground

Fish that were tagged and detected in the same season were excluded from the analysis due to a possible negative influence of the tagging procedure on their behaviour. Hence, fish tagged in 2014 were analysed in the spawning seasons of 2015 and 2016, and fish tagged in 2015 were analysed in the spawning season of 2016. Each fish recorded by the antennas was defined as a potential spawner. A properly installed antenna may achieve 97 ± 1.5% detectability of 32-mm tags, where missed detection may be the result of a high swimming speed and swimming in a group, or a parallel orientation of the tag to the antenna^50^. Therefore, it is possible for fish to leave the spawning ground without being detected lastly by antenna 1. Hence, we used merged detections by all antennas as a proxy for individual presence at the spawning ground and to avoid the misinterpretation of sequential records from antennas. Thanks to their active mating system where the fish frequently swam over the entire range of the spawning ground and do not occupy territories^32^, fish were frequently recorded by antennas when considered as present (see results above).

In the seasonal analysis, we tested for sex-specific differences in spawning effort and specifically whether males exhibit greater mate-finding effort and are seasonally protandric by analysing the follow variables: the detection frequency per hour, the hours and days detected at the spawning ground, and the first and last day of detection (on a scale, using the number of monitoring days in a given year). To simplify the tested variables, a single detection in an hour/day was defined as sufficient to count the fish as present in the given hour/day. The detection frequency per hour was calculated from the hours in which the individual was detected at the spawning ground. Statistical comparisons were conducted using a non-parametric one-sided Wilcoxon rank-sum test. Bonferroni corrections were applied to the estimated p-values.

#### Daily protandry, time of leaving, male length of stay and operational sex ratios

Daily protandry was defined as an earlier daily presence of males, on average, compared with the presence of females. To test the difference between male and female timing, we used the fish presence data. Individual fish was considered as present on the spawning ground when the time between two subsequent detections did not exceed one hour. A rank envelope test was used to identify the difference between male and female presence distributions^51^. A rank envelope test consists of computing simultaneous global envelopes for every functional value. Any departure of the data from the global envelope proves the rejection of the null hypothesis under global significance. Here, the null hypothesis was that both presence functions would follow the same distribution.

Asp presence had a clear bimodal pattern around sunset and sunrise (Fig. 3). Hence, we defined the two main spawning periods (peaking at sunset and sunrise) for use in GLM tests as the periods between the two points with the lowest abundance. Within these two periods (further referred to as evening and morning according to the peaks of activity), the times of arrival and leaving were defined as the times of first and last detection within the period range, respectively. Because migration peaked around sunset and sunrise and the day length changed during the spawning season, we expressed the arrival and leaving times as the difference from the time the sun set (sunrise for the morning peak). To test whether male length of stay depends on the number of females in the spawning ground, we used a Spearman rank-order correlation coefficient in order to eliminate the effect of distant measurements and to avoid the normality assumption. The number of hours that individual males were present was related to the number of females present in a given daily period. The analysis was performed separately for the evening and morning periods. In this and subsequent analysis, we used a subset of data with at least three females in the tested period to analyse a relatively representative estimate of reproductive period.

Finally, we tested whether the degree of protandry (differential value between individual male and average female arrival times) depends on the operational sex ratio in a given period using a GLM analysis, where fish ID was included as a random variable. Protandry was calculated for each male as the difference between his arrival time and the mean female arrival time in a given period. For the computation of operational sex ratio, we assumed that all fish present on the spawning ground are ready to mate. Operational sex ratio was computed as the fraction of the number of males and females present on the spawning ground within the given day period where these numbers were first corrected for the unequal number of tagged males and females arrived over the season and sex ratio in the population.

Statistical analyses were performed using Statistica software (Statistica, Inc., StatSoft, Tulsa, Oklahoma, USA) and R software version 3.2.3^52^.

## Electronic supplementary material

Seasonal arrivals and departures
