# Supplementary material for: Seasonal and daily protandry in a cyprinid fish
Source: Sci Rep. 2017 Jul 5;7:4737. doi: 10.1038/s41598-017-04827-x (PMC5498546; doi:10.1038/s41598-017-04827-x)
Supplement: Supplementary file 1 — Seasonal arrivals and departures [file 41598_2017_4827_MOESM1_ESM.pdf]

Seasonal and daily protandry in a cyprinid fish

Marek Šmejkal[a,b*], Daniel Ricard[a], Lukáš Vejřík[a], Tomáš Mrkvička[a,c], Lucie Vebrová[a,b], Roman Baran[a], Petr Blabolil[a], Zuzana Sajdlová[a], Ivana Vejříková[a], Marie Prchalová[a], Jan Kubečka[a]

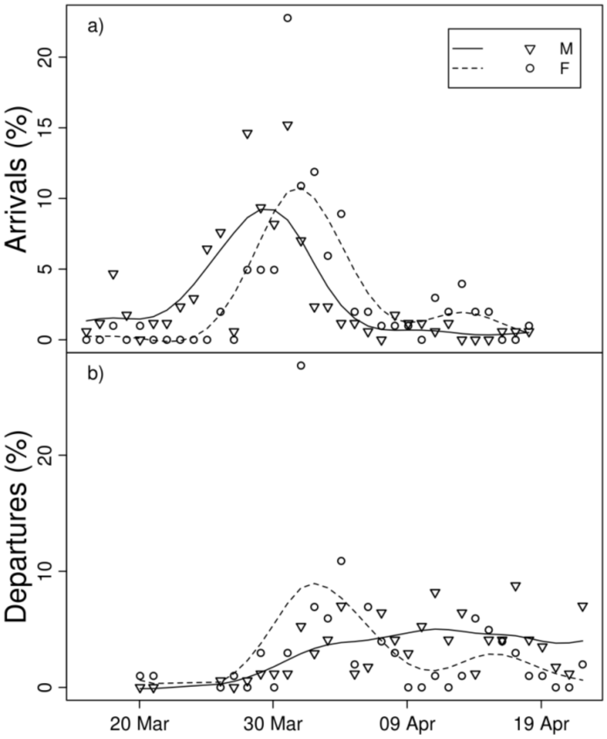

Supplementary figure 1: Arrival (a) and departure date (b) in 2016 plotted for females (F) and males (M) separately. Lines in panels (a) and (b) represent the percentage of arriving and departing individuals in given day. Open triangles and circles represent individual data points, from which lines were derived.
